# Supplementary figures and images for: vvv2_align_SE, vvv2_align_PE/vvv2_display: Galaxy-Based Workflows and Tool Designed to Perform, Summarize and Visualize Variant Calling and Annotation in Viral Genome Assemblies
Source: Viruses. 2025 Oct 17;17(10):1385. doi: 10.3390/v17101385 (PMC12567792; doi:10.3390/v17101385)

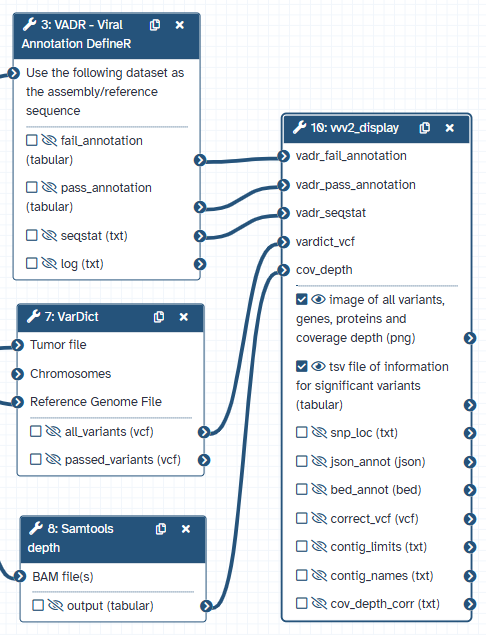

Supplement: Supplementary file 1 [file viruses-17-01385-s001.zip › SuppFig1_vvv2_display_brick_input_dep.png]

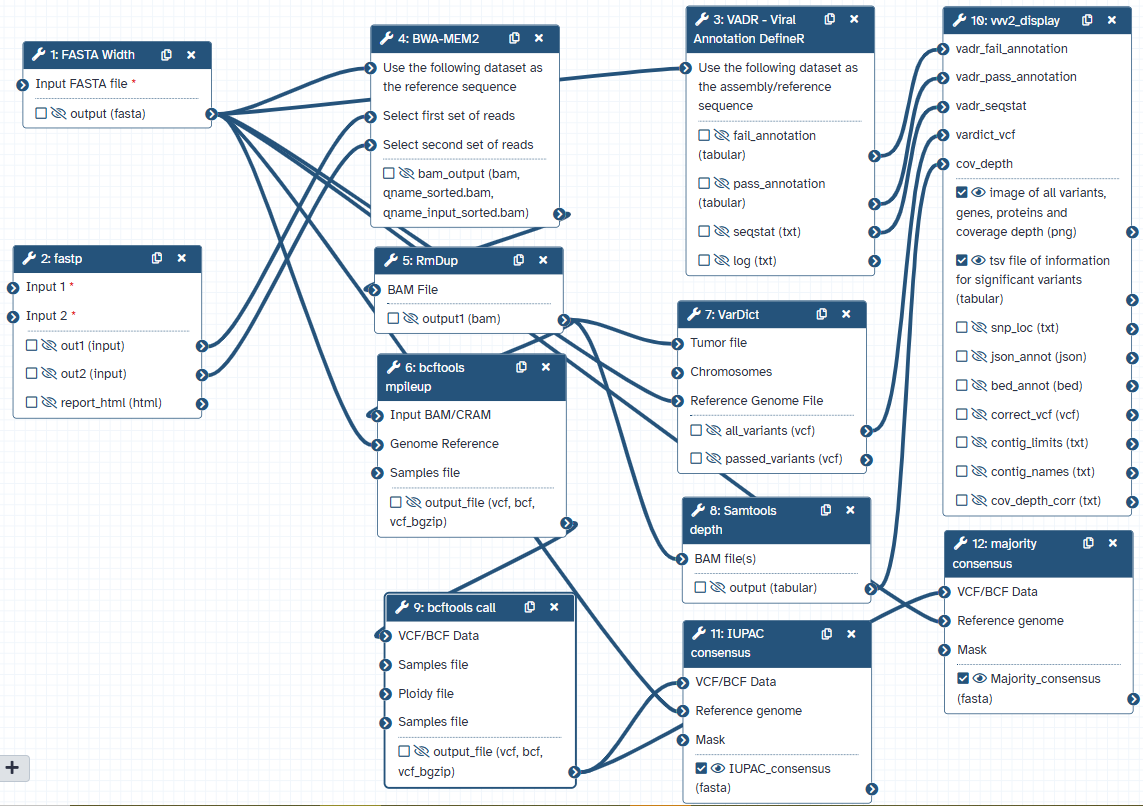

Supplement: Supplementary file 1 [file viruses-17-01385-s001.zip › SupplFig2_workflow_vvv2_align_bwamem_PE.png]
